# Does Scientific Evidence for the Use of Natural Products in the Treatment of Oral Candidiasis Exist? A Systematic Review

**DOI:** 10.1155/2015/147804

**Published:** 2015-03-26

**Authors:** Gabriela Lacet Silva Ferreira, Ana Luíza Alves de Lima Pérez, Ítalo Martins Rocha, Mayara Abreu Pinheiro, Ricardo Dias de Castro, Hugo Lemes Carlo, Edeltrudes de Oliveira Lima, Lúcio Roberto Castellano

**Affiliations:** ^1^Postgraduate Program in Dentistry, School of Dentistry, Universidade Federal da Paraíba, 58051-900 Joao Pessoa, PB, Brazil; ^2^Human Immunology Research and Education Group (GEPIH), Escola Técnica de Saúde da UFPB, Universidade Federal da Paraíba, 58051-900 Joao Pessoa, PB, Brazil

## Abstract

In view of the limitations of antifungal agents used in the treatment of oral candidiasis and the wide variety of natural products that have been studied as treatment of this disease, this systematic literature review proposed to evaluate whether scientific evidence attesting to the efficacy of natural products in the treatment of this disease exists. A systematic search in PubMed, MEDLINE, SciELO, Lilacs, and Cochrane Library databases was accomplished using the associations among the keywords Candida albicans, phytotherapy, biological products, denture stomatitis, and oral candidiasis in both English and Portuguese. Four independent observers evaluated the methodological quality of the resulting articles. Three studies were included for detailed analysis and evaluated according to the analysis protocol based on the CONSORT (Consolidated Standards of Reporting Trials) 2010 statement. The tested products were different in all studies. Two studies mentioned random samples, but no study described the sample allocation. No study mentioned sample calculations, a prior pilot study, or examiner calibration, and only one trial reported sample losses. Differences between the tested products and the methodological designs among these studies did not allow the existence of scientific evidence related to the effectiveness of these products for the proposed subjects to be confirmed.

## 1. Introduction

Oral candidiasis, which is produced by yeast of the genus* Candida*, is the mucocutaneous mycosis present in the oral cavity [1]. Generally, oral candidiasis affects users of complete upper dentures and is called denture stomatitis. Denture stomatitis is characterized by the presence of edematous and erythematous mucosa beneath an area covered by the prosthesis [2, 3].

*Candida albicans* is the most important microorganism in the pathogenesis of candidiasis and is present in the normal flora of the oral cavity. However, the transition from normal mucosal conditions to a parasitism situation may occur when an imbalance between host and fungus arises, which can lead to the onset of the oral candidiasis. The predisposing factors for oral candidiasis and denture stomatitis include systemic diseases, immune deficiencies, reduced salivary flow, broad-spectrum antibiotic usage, continuous prosthesis usage nightly, smoking, and poor oral and denture hygiene [4].

Although these diseases can be asymptomatic, some patients may experience discomfort such as swelling, pain, and burning sensations in the mouth [5], impairing the ingestion of liquids and food and, consequently, the quality of life of these patients [6]. Several commercially available antifungal agents are used to treat oral* Candida* infection, including nystatin, amphotericin B, clotrimazole, miconazole, itraconazole, fluconazole, and ketoconazole. However, despite their effectiveness, these drugs may produce adverse effects such as bitter taste, allergic reactions, and drug interactions [2, 3].

The development of natural products capable of clinical application is needed to create new strategies to control oral candidiasis because of the drawbacks and weaknesses of commercially available products. Natural products are promising therapeutic alternatives because they tend to display much smaller and lower intensity adverse reactions compared to allopathic drugs. Notably, the study of natural products can provide health professionals with alternative, feasible, and low-cost therapies for treating oral diseases [7].

Therefore, the use of medicinal plants and natural products for the treatment of these diseases has been extensively investigated; however, the scientific evidence from these studies has not yet been consolidated. Randomized clinical trials are the most suitable study design for providing evidence regarding the effects of an intervention study. However, the results of only one of these studies are not sufficient to clarify certain issues. In this sense, systematic reviews and meta-analyses are the most appropriate and current methods to summarize and synthesize evidence regarding the effectiveness and effects of interventions [3, 8].

Thus, the aim of this study was to use a systematic literature review to evaluate whether scientific evidence attesting to the efficacy of natural products in the treatment of oral candidiasis exists.

## 2. Materials and Methods

A systematic literature review was performed using the methodology proposed by Higgins and Green [9]. The screening and selection of articles adopted the following criteria.

*Inclusion Criteria*. We included studies in English, Spanish, and Portuguese that were randomized controlled trials and systematic reviews of all ages and both genders that examined products for use in dentistry based on natural substances with or without reduced clinical and/or microbiological signs and symptoms of oral candidiasis.

*Exclusion Criteria*. We excluded all studies that did not meet the inclusion criteria of this research that evaluated the associations of synthetic and natural products.

*Search Strategies*. The identification of articles was accomplished using a systematic search in the PubMed (National Library of Medicine), MEDLINE (International Literature on Health Sciences), SciELO (Scientific Electronic Library Online), Lilacs (Latin American and Caribbean Literature on Health Sciences), and Cochrane Library databases.

The search strategy in PubMed was performed based on the association of the following words using the search option “all fields”: (*Candida albicans* AND phytotherapy) OR (*Candida albicans* AND biological products) OR (stomatitis, denture AND phytotherapy) OR (stomatitis, denture AND biological products) OR (candidiasis, oral AND phytotherapy) OR (candidiasis, oral AND biological products). To refine the search, filters such as controlled trial, systematic review, and humans were used.

The search for articles was performed such that the greatest number of studies was found. The strategy used in the MEDLINE (search option “subject descriptor”), SciELO (search option “subject”), Lilacs (search option “all indexes”), and Cochrane (search option “title, abstract, and keywords”) databases was as follows: (*Candida albicans* AND phytotherapy) OR (*Candida albicans* AND biological products) OR (denture stomatitis AND phytotherapy) OR (denture stomatitis AND biological products) OR (oral candidiasis AND phytotherapy) OR (oral candidiasis AND biological products) OR (*Candida albicans* AND fitoterapia) OR (*Candida albicans* AND produtos biológicos) OR (estomatite sob prótese AND fitoterapia) OR (estomatite sob prótese AND produtos biológicos) OR (candidíase bucal AND fitoterapia) OR (candidíase bucal AND produtos biológicos).

All articles related to these word associations and published by May 2014 were selected for analysis. Four independent observers evaluated the methodological quality of the selected articles (the title and abstract) to verify whether these articles met the inclusion criteria. In cases where the data contained in the abstract were insufficient for determining the inclusion of the study, the full text was reviewed. After individual assessments, the examiners came to a consensus regarding the inclusion of studies for the evaluation of the full text.

Finally, the selected studies were screened using the Jadad scale [10], and those studies with scores greater than or equal to 3 were evaluated according to the analysis protocol based on the CONSORT (Consolidated Standards of Reporting Trials) 2010 statement [11].

Protocol followed by the examiners for the analysis of articles included in this systematic review is as follows:

1. preliminary analysis: title, primary author, country, language, journal, impact factor, and year of publication;
2. methodological review:
  (2.1) primary outcome of interest: with or without reduced clinical and/or microbiological signs and symptoms of oral candidiasis;
  (2.2) assessment of the quality of clinical trials: Jadad scale [10], with studies that obtained scores less than 3 being excluded from this review;
  (2.3) methodological design;
  (2.4) type of blinding and type of sample allocation;
  (2.5) profile, sample size, and sample size calculation;
  (2.6) loss of sample and reasons;
  (2.7) masking of product color, smell, and taste;
  (2.8) presence and characterization of placebo or control group;
  (2.9) comparison between control and experimental groups at the beginning of the study: description of groups to assess the equivalence between them at the initial phase;
  (2.10) quote of a pilot study;
  (2.11) quality of result measurement: inter- and intraexaminer calibration;
  (2.12) criteria used for clinical and/or microbiological evaluation for the disease diagnosis;
  (2.13) statistical analysis and significance level;
  (2.14) type of clinical trial: phase I, II, III, or IV according to Chalmers et al. [12];
3. analysis of intervention:
  (3.1) pharmaceutical form of the test product: gel, paste, or mouthwash;
  (3.2) product concentration;
  (3.3) dose range: amount and frequency per day and the time when the product is being used;
  (3.4) time of use (days or weeks);
  (3.5) clinical condition assessment intervals;
  (3.6) adherence to treatment, daily monitoring, and adverse effects (reports of discomfort caused by the product).
4. analysis of results: verification of accuracy according to the confidence interval and the sample size;
5. analysis of conclusions: determining whether conclusion meets the goals.

## 3. Results

**Figure 1:**
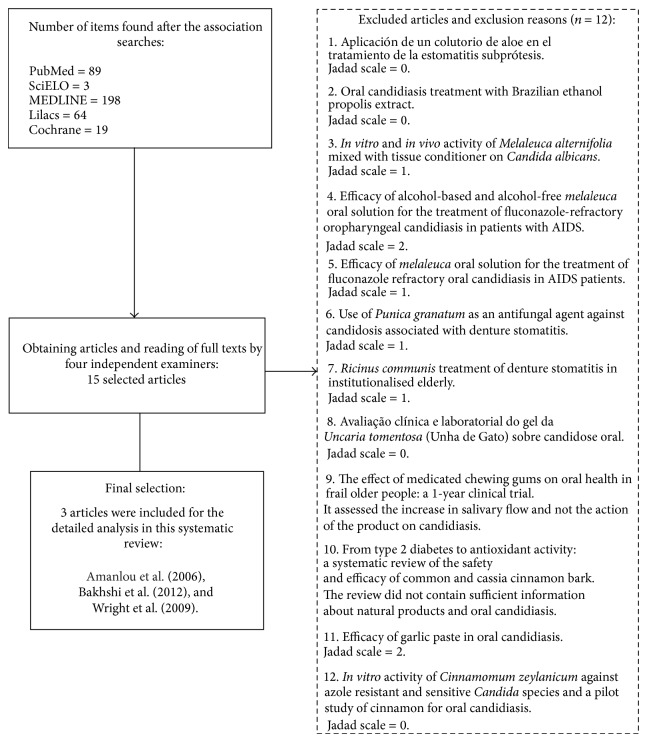
Flowchart of the search strategy.

According to the strategic search, 378 studies were found. After excluding repetitions, 301 different articles were identified (Figure 1). Of this total, fifteen articles met the inclusion criteria and were selected for further analysis. After careful analysis, three studies were considered of high importance and were included in this systematic review. The following three were the controlled clinical trials included in this analysis:

- treatment of oral thrush in HIV/AIDS patients using lemon juice, lemon grass (*Cymbopogon citratus*), and gentian violet [13];
- comparison of the therapeutic effects of an aqueous garlic extract and a nystatin mouthwash on denture stomatitis [3];
- miconazole gel compared with* Zataria multiflora* Boiss. gel in the treatment of denture stomatitis [2].

### 3.1. Data Description

The three trials were conducted in English (*n* = 3) in two countries, Iran (*n* = 2) and South Africa (*n* = 1). All three trials scored 3 on the Jadad scale (*n* = 3).

Table 1 presents data regarding the study design and characterization. All three trials were randomized (*n* = 3), and two were performed as a double-blind (*n* = 1) or triple-blind (*n* = 1) trial. The three studies described the sample profile; however, none of them mentioned the type of sample allocation.

One study reported follow-up losses; however, none of the studies mentioned conducting sample calculations or performing a pilot study or inter- and intraexaminer calibration. The three studies characterized the control group regarding the concentration of the product and the form of use. All articles provided the concentration of the test product, the quantity and time of use, and the intervals of clinical evaluation (Table 1).

Table 2 presents the data regarding the initial comparison between groups, the criteria used for the initial evaluation, and descriptions of the statistical analyses. All studies presented complaints of adverse effects and conclusions that corresponded to the study objectives (Table 2).

## 4. Discussion

Given the large amount of publications testing new products for clinical use, researchers, clinicians, and managers do not likely have access and time to evaluate all of these publications. Discerning and condensing all the information contained in these manuscripts to apply this knowledge to different clinical situations are even more difficult [14]. In this context, in dentistry, systematic reviews have been proposed for evaluating existing scientific evidence to respond to specific questions and to present the evidence in an accessible format.

The primary features of a systematic review are as follows: the creation of preestablished goals with inclusion and exclusion criteria for selecting studies, clear and reproducible methodology, systematic searches that provide access to the largest number of studies that meet the selection requirements, careful evaluation of the methodology and conclusions of the included studies, and organization and synthesis of results and conclusions [9] to minimize bias and to provide reliable results that support decision making [14].

Considering the limitations of commercially available antifungal agents for treating oral candidiasis, which involve increased fungal resistance [15], high cost, and adverse effects [2, 3] related to treatment, natural products have been investigated as important alternatives for the treatment of this pathology. The diversity of clinical and laboratory studies in the literature that have tested the different natural products raises this important question: is there clinical evidence for the use of natural products in the treatment of oral candidiasis?

To answer a clinical question, clinical trials are the studies of choice. The evaluation of a clinical trial includes careful methodological analyses of sample size, randomization, blinding, control usage, and sample losses [10].

The determination of sample size is an important part of the design of a clinical study because it attempts to eliminate both bias and predictable errors. A smaller sample than necessary can compromise the quality of the study, making understanding and inferring the results difficult; however, an extremely large sample may induce the existence of differences between groups when compared [16]. None of the studies included in this systematic review mentioned sample size calculations for sample determination.

The use of a control group is recommended to enable comparisons of test products preferably with the gold standard for treating the studied pathology. Thus, this review study included only controlled clinical trials.

Randomization and blinding are requirements cited in the literature [10] to assess the quality of clinical trials because randomization is a process in which each individual has the same chance to participate in one of the groups. Blinding prevents the researcher, the individual, or the statistician to influence the results [11]. Both requirements prevent errors and biases during the study and were considered in the screening of manuscripts by applying the Jadad scale. Many studies were excluded because they did not mention randomization and blinding in the methodological procedures. Although most studies have indicated the use of these resources, Wright et al. [13] described the randomization process but did not perform blinding. Bakhshi et al. [3] described the process of randomization and blinding of researchers and statisticians involved; however, these authors did not detail the process of randomization and the masking of product color, taste, and smell, which would confirm the blinding of study participants. Amanlou et al. [2] characterized the blinding process, including the masking of product, but only mentioned the sample randomization process without describing it.

Because clinical studies involving monitoring participant follow-up for a certain period are subject to withdrawal due to several factors, losses are expected and should be mentioned [17]. Only Wright et al. [13] mention this fact. Because the greater the follow-up loss, the larger the questions regarding the study validity due to the higher occurrence of systematic errors [18], these data may be frequently omitted.

Another important aspect that should be considered in studies involving the follow-up of participants is maintaining a daily personal contact or media that try to encourage adherence to treatment and the correct use of products. None of the three included studies reported this form of contact with participants; however, in the study by Wright et al. [13], this form of contact was assumed to be controlled because subjects were institutionalized and because the products were administered by trained nurses.

The three evaluated studies showed different forms of intervention, including amounts, usage frequency, and treatment duration. This difference can be attributed to the different nature of the products tested and forms of presentation. During the intervention period and even after its completion, clinical evaluations to monitor treatment progress, as well as the presence of adverse effects observed by participants, are extremely important. All studies have reported follow-up intervals and recorded adverse effects. Amanlou et al. [2] and Bakhshi et al. [3] conducted weekly meetings with individuals for this purpose. Wright et al. [13] mentioned personal contact every two days. These follow-up intervals are relevant because the greater the proximity to subjects, the smaller the chances of follow-up losses and the greater the maintenance and effectiveness of the interventions.

Because clinical examination is of paramount importance in the diagnosis of oral candidiasis, microbial examination becomes an important auxiliary method for its confirmation. However, several diagnostic and classification tools for this disease are available, and, in an attempt to better understand and to compare data from different studies, these criteria should be standardized. All studies mentioned the stage of clinical examination but different parameters for the measurement and classification of lesions. Only Amanlou et al. [2] used the mycological exam of mucosa and dentures for confirmation.

Statistical analysis is an important step in analyzing the results because it reduces the probability of events occurring randomly. The judicious choice of the statistical test increases the reliability and accuracy of results [19]. One of the items evaluated in the selected studies was the use of statistical tests and their properties to provide answers to the guiding questions of the study according to the types of variables involved, the number of groups, and the sample size. Among the three studies evaluated, only Bakhshi et al. [3] clearly showed that the tests used were able to evaluate the data obtained and to answer the study objectives. However, all studies have limitations for not presenting a review of the clinical significance because the statistical significance presents the possibility of the obtained differences being true, regardless of the clinical importance, as determined by clinical judgment [9, 20].

A major difficulty in comparing the studies included in this systematic review was the variety of natural products tested and the forms of presenting these products. Confirming the existence of scientific evidence for the treatment of oral candidiasis with natural products is difficult when few studies meet the inclusion criteria and most have large methodological differences, whether in study design or in choosing the test product, concentrations, and pharmaceutical forms. All selected studies differ regarding these criteria. Thus, further clinical trials that address the study products by standardized methodologies and that evaluate different usage periods and various concentrations should be performed.

## 5. Conclusion

Currently, affirming the existence of scientific evidence for the use of natural products in the treatment of oral candidiasis is not possible.

## Figures and Tables

**Table 1:** Methodological aspects, design and quality of studies, and characterization of groups.

Primary author	Study design and blinding type	Primary outcome	Sample allocation and profile	Sample loss and calculation	Test product masking	Pilot study and examiner calibration	Control group characterization	Test product pharmaceutical form, concentration, and usage time
Wright [13]	Randomized controlled clinical trial. Study was not blind.	Treatment of oral candidiasis: clinical regression of lesions.	90 patients with HIV/AIDS in a sanatorium. These patients were diagnosed with oral candidiasis and were not being treated for this purpose. Not mentioned: sample allocation.	The study began with 90 patients and finished with 52. Not mentioned: calculation.	There was no masking.	Not mentioned: pilot study and examiner calibration.	Gentian violet aqueous solution (0.5%). Painted on the inside of the mouth three times daily for 10 days.	Lemon juice (experimental treatment 1) for 10 days or until clinical cure; lemon grass (experimental treatment 2) for 10 days. Not mentioned: concentrations.

Bakhshi [3]	Controlled randomized double-blind clinical trial. Not mentioned: the blinding of study subjects.	Treatment of stomatitis under denture: clinical reduction of lesions after mouthwash.	40 institutionalized elderly subjects diagnosed with denture stomatitis. Not mentioned: sample allocation.	Not mentioned: calculation and loss.	Garlic aqueous solution and nystatin mouthwash were in similar bottles with the same shape, size, and color. Not mentioned: masking of smell, color, and taste.	Not mentioned: pilot study and examiner calibration.	Nystatin mouthwash (100,000 U/mL). Swish 20 drops for 60 seconds, three times a day.	Garlic aqueous solution (40 mg/mL) for 4 weeks.

Amanlou [2]	Randomized controlled triple-blind clinical trial.	Treatment of stomatitis under denture associated with *Candida*: reduction of clinical signs and symptoms and microbiological findings.	24 users of dentures diagnosed with denture stomatitis (clinical and microbiological), aged between 45 and 83 years. Not mentioned: sample allocation.	Not mentioned: calculation and loss.	Masking of smell, color, and taste as much as possible.	Not mentioned: pilot study and examiner calibration.	Miconazole 2% gel. Apply 2.5 mL on the base of the denture and place it in, four times a day for a period of 2 weeks.	*Z*. *multiflora* essential oil 0.1% gel for 2 weeks.

**Table 2:** Data collection, statistical analysis, results, and conclusions.

Primary author	Initial comparison between groups	Criteria for diagnosis	Follow-up treatment	Statistical analysis	Analysis of results and conclusions
Wright [13]	Age, gender, BMI, oral candidiasis scale on the day of admission, and number of days with symptoms were compared, and no difference between groups was observed.	Oral candidiasis was diagnosed and characterized from a 0 to 4 scale, where 0 represents no disease and 4 severe degree (oral thrush scale).	Once the patients were institutionalized, the treatment was controlled by nurses.	Data were gathered as ordinal data and analyzed by the following statistical tests: Fisher's exact test, chi-square test, and chi-square test with the continuity correction and the likelihood ratio. Level of significance: 95%.	Whether the sample was adequate is unknown because the author does not mention sample calculations. The quality of the analysis is doubtful due to the test selection. The choice of statistical test was inadequate for the ordinal data. The conclusion answers the aims.

Bakhshi [3]	The groups were compared regarding the methods used for cleansing dentures and the size of erythematous lesions present before treatment. No significant difference was observed between groups.	Diagnosis was established by measuring the length and width of erythematous lesions underneath the dentures by an oral medicine specialist using an oral caliper.	Not mentioned: daily monitoring to assess adherence to treatment and the correct use of the products.	ANOVA repeated measures + LSD post hoc test. Chi-square test is mentioned in the methodology but is not referred to in the results or in the analysis itself. The level of significance is not mentioned specifically. In the results, we find references for 99.9% and 99.99%.	The analysis is accurate because the tests chosen are suitable for the type of obtained data. However, whether the sample was adequate is unknown because the author does not mention sample calculations. The conclusion answers the aims.

Amanlou [2]	The groups were compared in terms of age, gender, history of systemic disease, and detection of *C*. *albicans* on denture surfaces and palatal sample. No significant difference was observed between groups.	Erythematous denture, covered palatal mucosa graded as moderate or severe. Diagnosed candidiasis was confirmed by microbiologic cultures from the palatal mucosa and from the denture surface.	There is no information regarding adherence to treatment or follow-up to control the proper use of medication.	Chi-square analysis, Student's *t*-test, and Mann-Whitney *U* test for independent samples. Level of significance: 95%.	Whether the sample was adequate is unknown because the author does not mention sample calculations. We cannot affirm the accuracy of the statistical analysis because the text does not mention a test to assess paired ordinal data and because the mentioned tests do not cover the entire analysis. The conclusion answers the aims.
